# Polyamines and α-Carbonic Anhydrases

**DOI:** 10.3390/molecules21121726

**Published:** 2016-12-15

**Authors:** Andrea Scozzafava, Claudiu T. Supuran, Fabrizio Carta

**Affiliations:** 1 Laboratorio di Chimica Bioinorganica, Università degli Studi di Firenze, Rm. 188, Via della Lastruccia 3, 50019 Sesto Fiorentino, Florence, Italy; andrea.scozzafava@unifi.it; 2 NEUROFARBA Department, University of Florence, Sezione di Scienze Farmaceutiche, Via Ugo Schiff 6, 50019 Sesto Fiorentino, Florence, Italy

**Keywords:** polyamines, spermine, CA inhibitors

## Abstract

Natural products represent a straightforward source for molecular structures bearing a vast array of chemical features and potentially useful for biomedical purposes. Recent examples of this type include the discovery of the coumarins and the polyamine natural products as atypical chemotypes for the inhibition of the metalloenzymes carbonic anhydrases (CAs; EC 4.2.2.1). CA enzymes are established pharmacological targets for important pathologies, which, among others, include glaucoma, hypoxic tumors, and central nervous system (CNS)-affecting diseases. Moreover, they are expressed in many bacteria, fungi and helminths which are the etiological agents of the majority of infectious diseases. In this context, natural products represent the ideal source of new and selective druggable CA modulators for biomedical purposes. Herein we report the state of the art on polyamines of natural origin as well as of synthetic derivatives as inhibitors of human CAs.

## 1. Polyamines in Biology

Polyamines are defined as low-molecular-weight molecules, bearing multiple nitrogen atoms. In nature they are abundantly present both as unconjugated derivatives or conjugated to phenolic acids and biological macromolecules such as DNA/RNA complexes [1,2,3,4,5,6,7,8]. The polyamines’ unique structural and electronic features are responsible for their multiple roles and modes of action, which are mainly governed through ionic interactions of the spaced positive charges [4]. It was demonstrated that the exposure of polyamines to isolated strands of DNA or in the form of chromatin induced condensation of the genetic material [9]. In double-strand DNA, polyamines bind both to the minor and the major grooves and additional electrostatic interactions can also occur with the polyphosphate DNA scaffold [9]. The induced conformational changes to the nucleic acids might be considered as a temporary macromolecular stabilization or as an additional fine-tuning of the gene regulation machinery system. In thermophilic bacteria polyamines are implicated in the stabilization of DNA and RNAs at high temperatures [1].

The biosyntheses of the polyamines are precisely regulated as they play key roles within the eukaryotic cells’ life cycle, such as regulation of the ion channel and cell signaling, stabilization of biological macromolecules and gene regulation [5].

**Figure 1:**
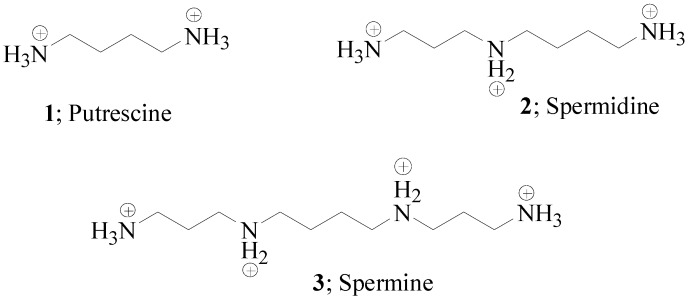
Chemical structures of polyamines **1**–**3** at physiological pH.

Herein we report on polyamines of natural origin, such as putrescine **1**, spermidine **2** and spermine **3** (Figure 1) as well as their structurally related derivatives of synthetic origin [10] or natural sources [11] which have, until now, been investigated as human (h) Carbonic Anhydrase (CA) inhibitors.

Putrescine **1**, spermidine **2** and spermine **3** represent the main pool of polyamines in the eukaryotic cells and are all derived from decarboxylation reactions of ornithine or *S*-adenosyl-methionine amino acids by the enzyme ornithine decarboxylase (ODC) [8].

The polyamines’ biosynthetic events are particularly accelerated in fast growing cells, such as in tumors, as demonstrated by the high levels of the proto-oncogene ODC in animal tumor models or in breast and colon cancer specimens [12]. Since ODC catalyzes the rate-limiting step of the polyamines’ biosynthesis, it is a marker of particular relevance in tumorigenesis [8].

**Scheme 1:**
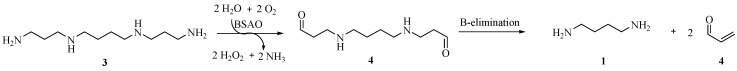
Oxidative deamination of spermine **3** catalyzed by the bovine serum amine oxidase (BSAO) [13].

In cells, the higher-molecular-weight polyamines, such as spermine **3**, are dismantled to smaller constituents through an oxidative deamination process. Such a concept was proved by means of the experiment reported in Scheme 1 [13].

The polyamine spermine **3** was subjected to oxidation at the terminal ends by means of the bovine serum amine oxidase (BSAO, EC 1.4.3.6) to generate ammonia and hydrogen peroxide. Then the dialdehyde intermediate **4** underwent a β-elimination to the shorter polyamine putrescine **1** and acrolein **4**. The overall result of the high-molecular-weight polyamines’ catabolism, such as that of spermine **3**, is the formation of highly cytotoxic metabolites, which in turn are responsible for triggering cell death via apoptotic or non-apoptotic pathways [13]. It is clear that the polyamines’ transformation into biological systems is also linked to the regulation of cell proliferation and death events.

**Figure 2:**
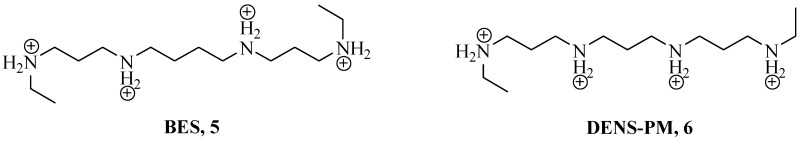
Chemical structures of BES (*N*1,*N*12-diethyl spermine tetrahydrochloride) **5** and DENS-PM (*N*1,*N*11-diethyl norspermine tetrahydrochloride) **6**.

This makes the polyamines a valid and alternative tool for the design of a new class of antitumoral drugs. Encouraging results on this topic were obtained with the *N*-alkylated spermine analogues reported in Figure 2, which act by mimicking the structure of the biogenic polyamines, without any induction of their biological functions [14].

Experiments on in vivo tumor models revealed that the spermine synthetic derivatives *N*1,*N*12-diethyl spermine tetrahydrochloride (BES) **5** and *N*1,*N*11-diethyl norspermine tetrahydrochloride (DENS-PM) **6** stopped cellular proliferation, induced apoptosis and showed low toxicity. From the biological viewpoint, the cell deaths induced by the use of polyamine synthetic derivatives are ascribed to a multi-targeting effect, such as gene and ionic channel deregulations, destabilization of biological macromolecules (i.e., DNA and RNA), as well as induced alteration of the functionality of some cellular organelles. In this context, mitochondria are of particular interest as they lack the enzymatic tools for the biosynthesis of polyamines but instead possess efficient and specific polyamine transportation systems [15].

## 2. Polyamines and CAs

**Figure 3:**
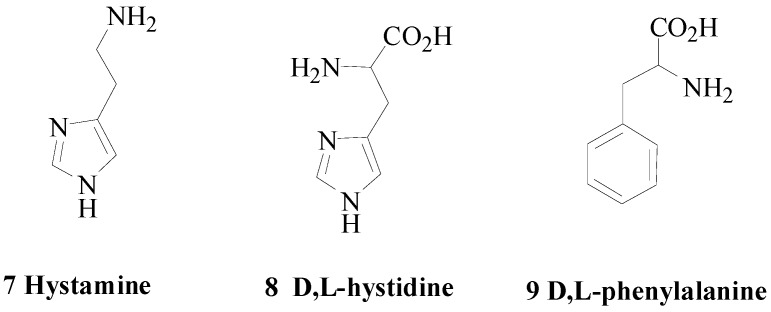
Classical carbonic anhydrase activator (CAAs).

Usually small molecules possessing a terminal amino end enhance the catalytic activities of the metalloenzymes carbonic anhydrases (CAs; CAs; EC 4.2.2.1). The classical CA activators (CAAs) include biogenic amines such as hystamine or amino acids of the type reported below (Figure 3) [16,17,18,19,20].

**Scheme 2:**
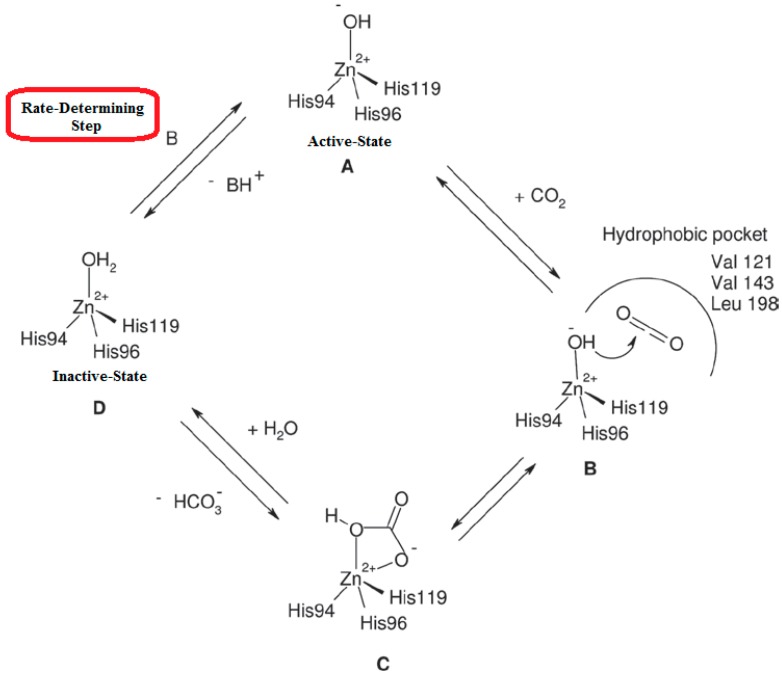
Catalytic cycle of hCAs [21].

From the mechanistic viewpoint, the CAAs act at the last step of the CA catalytic cycle (conversion of D to A in Scheme 2), which is also the rate-determining one.

**Figure 4:**
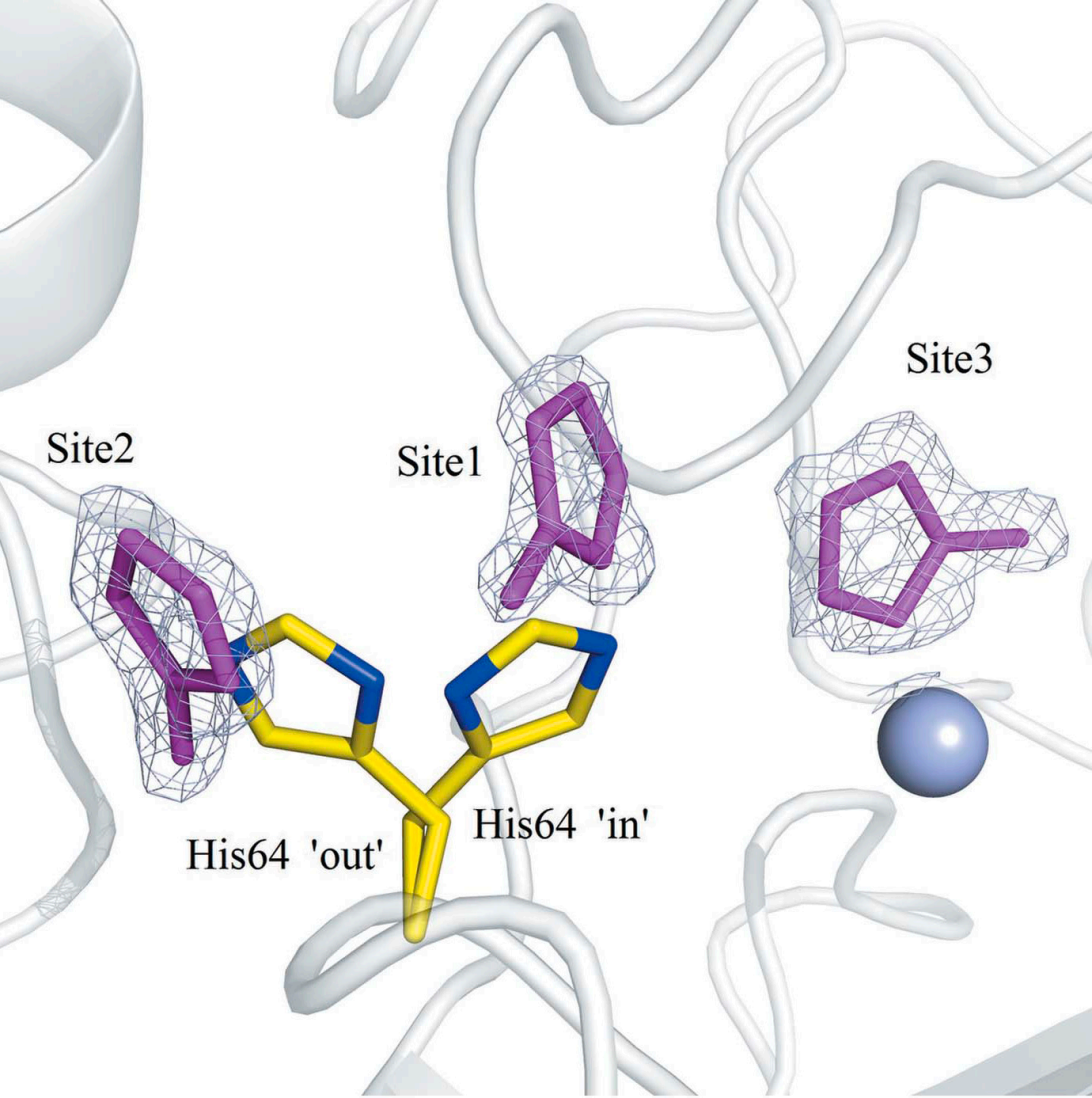
Binding sites (sites 1–3) of 4-methylimidazole (4MI) within and near the active site of His64A hCA II. The 2Fo Fc electron-density maps are contoured at 1.2 σ. His64 from wild-type CA II (PDB entry 3ks3 [23]) is shown as yellow sticks to represent the ‘in’ and ‘out’ conformations [22].

Among the α-CAs, the deprotonation of D to restore the enzyme to the active state A is supported by a histidine residue (His64 according to the h CA II numbering), which is located at the middle of the enzymatic cavity. Such a residue swings back and forth between two opposite conformations, thus facilitating the extrusion of protons to the external medium from the inner enzymatic cavity (Figure 4) [22,23].

CAAs enhance the proton extrusion rate by creating additional hydrogen nets through their protonatable amino functionalities. Thus, the enzyme is catalytically more efficient in the hydration of the natural ligand carbon dioxide [16,17,18,19,20,21,22,23].

The typical structural features associated with CAAs are a proper fitting of the ligand within the enzymatic cleft as well as the presence on it of protonatable moieties with p*K*_a_ values spanning between 6.5 and 8.0 [16]. Thus, it is expected that the polyamines act as CAAs.

**Figure 5:**
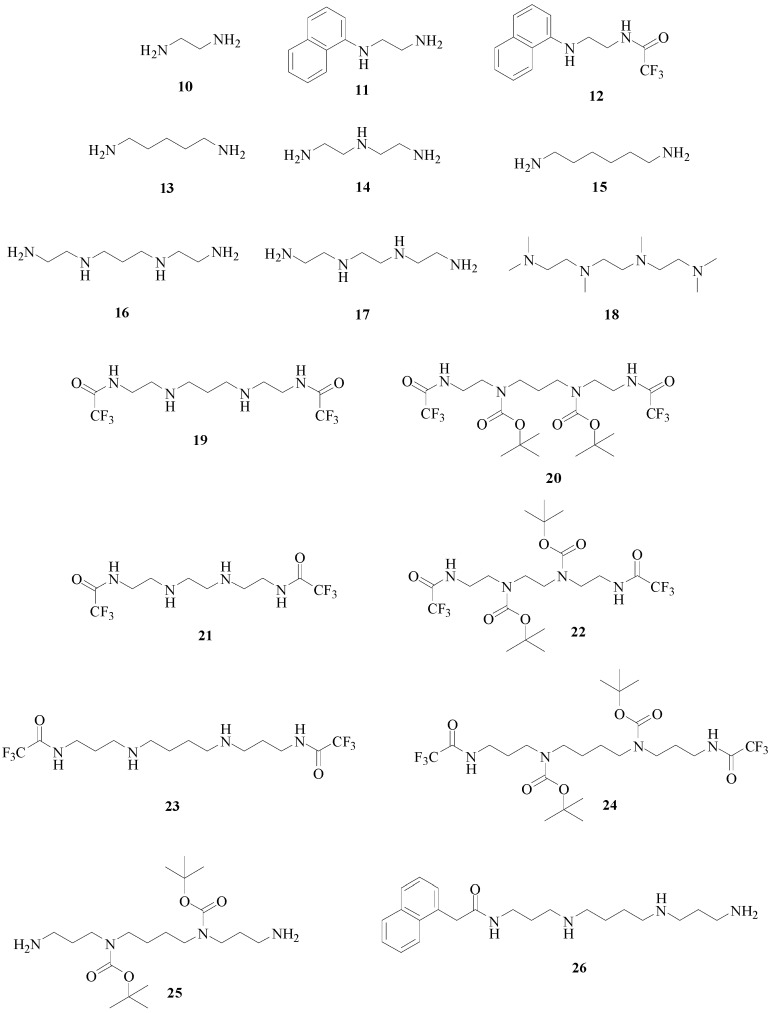
Series of synthetic polyamine derivatives **10**–**26** tested against the α-CAs [10].

We reported for the first time a series of biogenic polyamines, including spermidine **2** and spermine **3** as well as their synthetic derivatives (Figure 5), and their kinetic properties on human (h) and murine (m) catalytically active CAs (Table 1) [10].

Surprisingly, the reported compounds **2**, **3** and **11**–**26** did not show any activation properties towards the CAs considered. Instead they were inhibitors with K_I_ values between the nano- to the millimolar range. The only exception was represented by ethylenediamine **10** as any appreciable enzymatic modulation was detected (data not reported). All the in vitro kinetic results obtained were compared with those of established Carbonic Anhydrase Inhibitors CAIs, such as acetazolamide (**AAZ**) and phenol.

Among the biogenic polyamines considered, spermine **3** showed the most relevant CA inhibitory profile. Indeed, **3** was a weak inhibitor of the physiologically abundant hCA isoforms I and II (K_I_ 231 μM and 84 μM, respectively) and a rather good inhibitor of the mitochondrial hCAs VA and B (K_I_ 0.84 and 0.83 μM, respectively) of the secreted hCA VI (K_I_ 0.99 μM) and the CNS-dominant isoforms hCA VII and XIV (K_I_ 0.71 μM and 0.86 μM, respectively). Interestingly spermine **3** was revealed to be an efficient inhibitor of the membrane-bound hCA IV with a K_I_ value of 10 nM.

Spermidine **2**, the shorter biogenic analogue of **3**, acted preferentially on the hCA IV (K_I_ 0.112 μM) but also strongly inhibited all the remaining CA isoforms (K_I_ 1.0–11.6 μM), with the exception of the tumor-associated hCA XII (K_I_ 44.1 μM).

Reduction of the alkyl chain length, such as in **13**–**15**, resulted in a general decrease of the CA inhibitory activity, with the triamine **14** as the weakest in the series. Even so, the kinetic profile for **13** and **15** is an analogue to the parent biogenic polyamines spermidine **2** and spermine **3**, having hCA I and II as less inhibited among all the tested isoforms.

Direct Structure-Activity-Relationship SAR between the CA inhibition and the chain length is also evident when comparing the kinetic data of the *N*-polysubstituted spermine derivatives **23**–**25** with the corresponding shorter analogues **19**–**22**. A remarkable drop in the inhibitory activities is registered as the alkyl chain is reduced.

Another critical factor which modulates the CA inhibitory activity is represented by the number of the amine functionalities and their degree of substitution. Herein, all reported compounds have at least two nitrogen atoms, up to a maximum of four. When the chain length is kept constant and a -CH_2_- is substituted by nitrogen, such as in the conversion of **13** to triamine **14**, the CA inhibition drops.

As far as it concerns the substitution at the nitrogen, a good example is offered by the polyalkylation of trien **17** to give **18**. In this case, a two-orders-of-magnitude improvement for the CA inhibitory activity is observed in all the isozymes with the exception of hCA I. In general, all the data demonstrated that polyamines functionalized at the nitrogen atoms preserve a good CA inhibitory activity when at least a free terminal amino end is preserved. This is not the case of the spermine derivative **23** which has both the primary amino ends protected with trifluoroacetate groups. In fact, the CA inhibitory activity of **23** is maintained when compared to the parent spermine **4** and even ameliorated as for hCAs I–III.

Interestingly, the selective functionalization at one end enhances the CA inhibitory activity as demonstrated by the commercially available spermine derivative **26** and even more clearly by **11**. The introduction of the naphthyl group (compound **11**) to the inert ethylenediamine **10** restored the CA inhibitory activity which gets attenuated when the latest amino terminal is functionalized with the trifluoroacetate group (compound **12**). The introduction of bulky functionalities inevitably spoiled the CA inhibitory properties as clashes within the enzymatic cavity might occur (compounds **20**, **22**, **24** and **25** in Table 1).

**Figure 6:**
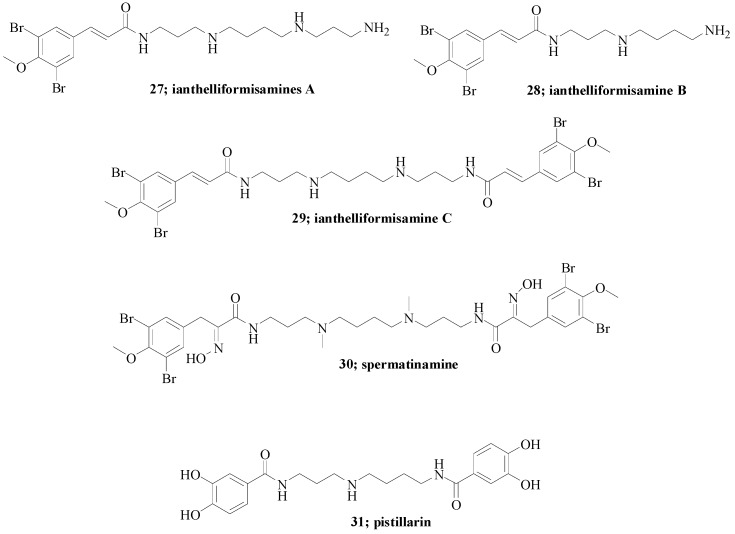
Structures of spermidine secondary metabolites **27**–**31** [11].

Another contribution in the field was from us in collaboration with the Davis Group at Eskitis on the series of spermidine secondary metabolites **27**–**31** (Figure 6) [11].

Anthelliformisamines A–C **27**–**29** were isolated from the marine sponge *Suberea* *ianthelliformis* [24], spermatinamine **30** from the sponge *Pseudoceratina* sp. and pistillarin **31** from fungal species such as *Penicillium bilaii* [25], *Gomphus floccosus* [26], *Clavariadelphus pistillaris* [27], and *Ramaria* species [27,28]. They were all previously considered for their antibacterial [24], antimalarial [28] and anticancer properties [29].

Table 2 reports the kinetic data of compounds **27**–**31** against the physiologically relevant hCAs I, II, IV, IX and XII [11].

Data reported in Table 2 revealed that compounds **27**–**31** showed similar inhibition potencies against the abundantly expressed hCAs I and II, comparable to that of spermidine **2**. As for the tumor-associated hCA CA IX and XII, **27**–**31** showed K_I_ values spanning between 0.2 and 4.21 μM; thus, they were more potent when compared to spermidine **2** and spermine **3** (Table 2). The only exception was spermatinamine **30** (K_I_ > 20 μM). The extracellular bound CA IV and XIV were weakly inhibited by **27**–**31**, in comparison with spermidine **2** and spermine **3** (Table 2).

Even if an exhaustive SAR was not feasible, it may suggested that many structural and electronic factors strongly affect the binding of such molecules with the CA enzymes.

## 3. Crystallographic Investigation

In order to reveal the binding mode of the polyamines as CAIs, a crystallographic investigation of spermine **3** in an adduct with the hCA II at a resolution of 2.0 Å was conducted. As for the kinetic experiments, the crystallographic investigation was conducted at pH 7.4, which means spermine **3** is fully protonated [10].

**Figure 7:**
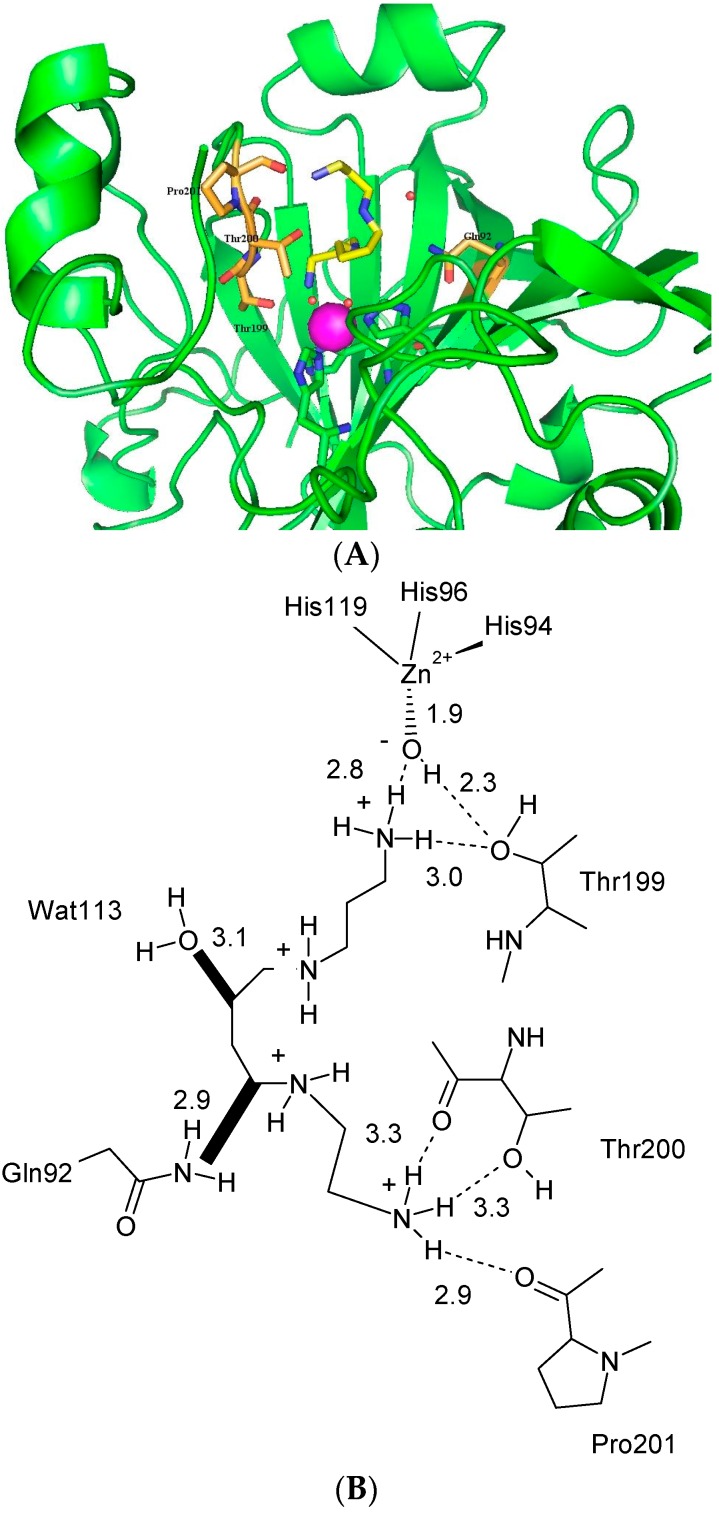
(**A**) Detailed interactions in which spermine (in yellow) participates when bound to the hCA II active site. The zinc ion (violet sphere), the water molecule coordinated to it (small red sphere), and its three protein ligands (His94, 96, and 119, in green), as well as amino acids involved in the binding of spermine **3** (Thr199, Thr200, Pro201 and Gln92, in gold), are also shown. The protein scaffold is in green (ribbon diagram); (**B**) Schematic representation of interactions in which spermine **3** (as tetracation) participates when bound to the hCA II active site. Figures represent distances (in Å). Hydrogen bonds are represented as dashed lines. In bold two clashes are shown involving some carbon atoms (C5 and C7) of the spermine **3** scaffold with a water molecule (Wat113) and Gln92. The non-protein zinc ligand is represented as a hydroxide ion, which should be the preponderant species at the pH at which the experiments were done (7.5). PDB accession code: 3 KWA [10].

The electron density maps clearly show the enzymatic cavity occupied by a single molecule of the inhibitor, which is stabilized in a coiled conformation (Figure 7) [10].

One ammonium terminal moiety of polyamine **3** is deeply buried into the enzymatic cavity and strongly interacts with the zinc-bound hydroxide (Figure 7A) in a fashion resembling the phenol–hCA II adduct binding mode (Figure 7B). In fact, one of the ammonium protons strongly interacts (2.8 Å) via the hydrogen bond with the zinc-bound hydroxide and another with the -OH (3.0 Å) of the conserved amino acid Thr199. The phenol–hCA II adduct differs at this point as the Thr199 participates in the phenol stabilization through its amidic -NH- (Figure 7B) [18]. The aliphatic carbon chain of spermine **3** participates on the adduct stabilization by making various Van der Waals interactions. Of particular note are the clashes the central alkyl section of spermine **3** retains with the water molecule 113 (3.1 Å) and the amide nitrogen of Gln92 (2.9 Å). The presence of such steric clashes might explain the low inhibitory activity of spermine **3** for hCA II (K_I_ 84 μM). The outer ammonium end is involved in a net of strong hydrogen bonding with Thr200 and Pro201, as shown in Figure 7A.

## 4. Conclusions

Polyamines revealed a completely unexpected behavior when tested for their CA activity. They possess good hCA inhibitory properties with K_I_ values spanning from the nano- to the millimolar range.

SAR revealed that many aspects account for the polyamines’ CA activity such as the chain length, the number of nitrogen atoms, and the number of functional groups introduced, as well as their electronic features.

Co-crystallographic experiments clearly demonstrate that polyamines bearing at least a terminal amino moiety interact within the CA enzymatic cavity in a very different manner when compared with known CA inhibitors such as the sulfonamides (or their bioisosters) [30], coumarines [31,32] or phenols [33]. Specifically spermine **3** adopts a helicoidal conformation within the enzymatic cavity and the buried ammonium end strongly interacts with the zinc-bound hydroxide through a net of hydrogen bonds. Moreover, additional hydrogen bonds, as well as multiple, weak Van der Waals contacts, contribute to the adduct stabilization.

The discovery of the polyamines as CA inhibitors opens innovative perspectives for the development of new lead compounds with modulating properties towards the CA enzymes.

## Figures and Tables

**Table 1:** Human (h)CA I–XII, XIV and murine (m)CA XIII and XV inhibition data with polyamines **2**, **3** and **11**–**26**. Data relative to ethylenediamine **10** are not shown [10].

Entry	K_I_ (μM) *												
	hCA I	II	III	IV	VA	VB	VI	VII	IX	XII	XIII	XIV	XV
**2**	1.40	1.11	11.5	0.11	1.22	1.44	1.41	1.23	1.37	44.1	11.6	1.0	10.0
**3**	231	84	167	0.01	0.84	0.83	0.99	0.71	13.3	27.6	22.6	0.86	74
**11**	>500	103	0.42	0.058	0.048	0.061	0.64	0.36	0.51	0.38	0.62	0.59	0.57
**12**	>500	121	128	12.3	106	107	109	1.24	12.2	21.5	127	34	110
**13**	13.3	11.0	0.50	0.052	0.044	0.54	0.74	0.42	0.38	0.45	0.63	0.50	0.65
**14**	415	118	117	116	110	11.0	11.5	12.1	10.6	11.4	11.5	10.1	105
**15**	12.6	34.4	0.60	0.45	0.61	0.58	0.72	0.44	0.41	0.37	0.69	0.64	0.66
**16**	115	75	63	44	50	59	53	58	48	68	66	36	66
**17**	100	64	48	35	38	49	43	45	39	57	52	12.1	59
**18**	>500	11.2	0.52	0.053	0.047	0.71	0.78	0.73	0.31	0.52	0.58	0.74	0.76
**19**	122	112	96	108	62	54	156	108	117	112	131	125	104
**20**	>500	>500	>500	309	416	401	>500	>500	>500	>500	>500	>500	>500
**21**	>500	>500	>500	>500	>500	>500	>500	>500	>500	>500	>500	>500	>500
**22**	>500	>500	>500	>500	>500	>500	>500	>500	>500	>500	>500	>500	>500
**23**	136	11.3	11.5	0.116	1.26	1.05	1.10	1.09	11.4	10.2	12.6	6.7	110
**24**	>500	107	112	104	125	103	104	107	124	175	179	85	>500
**25**	137	110	132	103	131	107	114	108	144	165	136	115	236
**26**	12.3	1.13	11.6	0.018	1.03	1.05	0.11	0.10	0.12	0.19	10.2	1.03	0.78
**AAZ**	0.25	0.012	200	0.074	0.063	0.054	0.011	0.0025	0.025	0.0057	0.017	0.041	0.072
**Phenol**	10.2	5.5	2.7	9.5	218	>500	208	>500	8.8	9.2	>500	11.5	10.5

* From three different assays, errors ±5%–10% of the reported value, CO_2_ hydrase, stopped-flow assay.

**Table 2:** Human (h)CA I, II, IV IX and XII inhibition data with polyamines **27**–**31** in comparison with spermidine **2**, spermine **3** and a classical CAI acetazolamide (**AAZ**) [11].

Entry	K_I_ (μM) *					
	hCA I	II	IV	IX	XII	XIV
**2**	1.40	1.11	0.11	1.37	44.1	1.0
**3**	231	84	0.01	13.3	27.6	0.86
**27**	1.76	0.41	6.72	0.20	2.81	2.12
**28**	0.77	0.37	9.10	0.35	3.48	2.28
**29**	0.86	0.35	9.08	0.27	3.50	6.96
**30**	0.85	0.48	>20	0.34	>20	2.72
**31**	0.79	0.34	7.03	0.36	4.21	1.52
**AAZ**	0.25	0.012	0.074	0.025	0.006	0.041

* From three different assays, errors ±5% of the reported value, CO_2_ hydrase, stopped-flow assay.
